# Astrocyte-Derived Extracellular Vesicles and the Evolution of Neural Complexity: Perspectives on Vesicle-Mediated Neuron–Glia Communication

**DOI:** 10.1007/s12035-026-06214-y

**Published:** 2026-09-29

**Authors:** Katarzyna Ciuba

**Affiliations:** Dioscuri Centre for Chromatin Biology and Epigenomics, Nencki Institute of Experimental Biology, Polish Academy of Sciences, Warsaw, Poland

**Keywords:** Astrocyte-derived extracellular vesicles, Intercellular communication, Astrocyte evolution, Brain intercellular signaling

## Abstract

The classic view of neural communication occurring exclusively via synapses has been challenged by growing evidence that glial cells, in particular astrocytes, play an active role in shaping neural circuits. Astrocytes impact neural networks via distinct mechanisms, some of them remaining elusive. Here, we discuss the role of astrocyte-derived extracellular vesicles (ADEVs) and their possible functions in complementing intercellular communication in parallel with synaptic communication and direct gliotransmission. Because astrocytes have undergone substantial evolutionary expansion and diversification in mammals, we also draw the perspective that frames ADEVs within an evolutionary context. We posit that ADEV-mediated signaling may represent an additional candidate axis of astrocyte–neuron communication and discuss testable predictions this hypothesis generates.

## Introduction

The evolutionary development of the human brain has endowed our species with distinctive cognitive abilities, such as self-awareness, advanced communication, speech, and abstract thinking. Understanding the biological mechanisms underlying these newly acquired skills is one of the most intriguing questions of neurobiology. So far, the majority of efforts have focused on neurons, considering them as the main drivers of brain evolution [1–3]. However, research over the past few decades has demonstrated that astrocytes, the predominant type of glial cells in the brain, also play a vital role in shaping cognitive functions by actively regulating synapse formation, maintenance, and pruning [4, 5]. The substantial morphological and functional advancements of human astrocytes suggest that these cells are important evolutionary targets involved in the development of higher-level brain functions.

The development and maintenance of refined cognitive skills requires precise communication both between brain cells and between the cells and their extracellular environment. Extracellular vesicles (EVs), small lipid-coated particles, act as “molecular messengers”, which are one of the means that facilitate the exchange of biological information. Produced by virtually all cell types in the body, EVs carry distinct cargo that can be transferred between cells over a range of distances, from the immediate pericellular space to long-range communication within the organism, that in the CNS is primarily mediated by cerebrospinal fluid and the circulation, as EVs can also cross the blood-brain barrier (BBB) [6].

Astrocyte-derived extracellular vesicles (ADEVs) are implicated in diverse processes, ranging from facilitating neuroprotective roles of astrocytes [7] to participation in regulation of synaptogenesis, synaptic activity, and plasticity [8]. ADEVs cargo varies in inflammatory [9] and pathological conditions [10]. Currently, it remains elusive whether the evolutionary expansion of astrocytes could be correlated with ADEV-mediated communication in the human brain.

Astrocytes are the most abundant macroglial cells in the brain and they play versatile roles in the CNS. Astrocytes participate in numerous homeostatic processes, like maintenance of the optimal concentration of ions and nutrients in the brain parenchyma and support of the blood-brain barrier. Further, astrocytes keep the physiological level of neurotransmitters by taking up their excess and by supplying their precursors to neurons [4]. Although historically perceived as purely housekeeping cells, recent data firmly established that through impacting neurogenesis, synaptogenesis, synaptic activity, and synaptic transmission, astrocytes contribute to the regulation of higher-level brain functions [5, 11–15].

Here, we propose and attempt to evaluate a hypothesis that ADEVs constitute a candidate cargo-based communication mechanism that extends astrocytic regulatory influence within the brain. Building on this, we propose that evolutionary diversification of astrocytes may have altered the functional role of ADEVs, potentially adding a complementary mode of intercellular molecular communication in the increasingly complex brain.

## Astrocyte Functional and Evolutionary Diversification

### Multitasking Astrocytes

Astrocytes exhibit a high degree of functional specialization, ranging from basic brain homeostasis maintenance to fine-tuned control of neuronal function, metabolic coupling, and calcium signaling. Astrocytes actively participate in regulating synapse transmission within the framework of tripartite synapse [14]. Astrocytic projection leaflets enwrap closely the pre- and postsynaptic neuronal terminals, enabling sensing and regulating the synaptic activity [16]. Astrocytes regulate neurotransmitter concentration at the synapses by taking up their excess, as well as release gliotransmitters that can further impact the synapse function [17–19]. This intercellular communication is central to the proper function of neural circuits [20]. Apart from involvement in synaptic regulation, astrocytes play a crucial role in maintaining metabolic coupling between neurons and glia by, among others, metabolizing glucose and supplying neurons with lactate, enabling activity-dependent energy distribution and coordinating metabolic homeostasis [21–23]. Further, astrocytic networks interconnected via gap junctions can distribute metabolites over larger distances, supporting neuronal populations beyond local synaptic environments [24, 25].

In contrast to neurons, astrocytes are not electrically excitable, yet they are actively communicating via intracellular calcium (Ca^2+^) transients that encode information on the state of local synaptic environment and translate it into downstream glial responses [26]. Ca^2+^ transients are actively regulated events that respond to such cues as synaptic glutamate or ATP release that impact synaptic function and overall neural network dynamics [27]. Moreover, astrocytic Ca^2+^ signaling is propagated through the astrocytic network in the form of intercellular waves, converting a local synaptic information into a spatially extended response that exceeds synaptic wiring coordination [28, 29].

Beyond synaptic and metabolic regulation, astrocytes are integral to neurovascular function. Astrocytic endfeet enwrap cerebral blood vessels and contribute to the formation and maintenance of the BBB. Moreover, astrocytes maintain the neurovascular coupling necessary for adjustments of local blood flow to neuronal activity [30, 31]. Further, astrocytes participate in perivascular fluid drainage by supporting clearance of interstitial solutes from the brain parenchyma, and contribute to neuroimmune regulation at the neurovascular interface [32]. These vascular and fluid-drainage roles are increasingly recognized as integral to how astrocytes support neuronal homeostasis, alongside their synaptic and metabolic functions.

### Expansion and Diversification of Astrocytes in Mammals

Astrocytes have undergone significant evolutionary expansion and diversification during mammalian brain evolution. This is especially pronounced in human astrocytes that exceed their rodent and non-human primate (NHP) counterparts in size, complexity and territorial coverage area [33]. Astrocyte expansion is genetically encoded: the same size and complexity differences are recapitulated in iPSC-derived astrocytes of human, chimpanzee, and macaque generated in vitro, alongside with activation of pathways associated with increased exosome biology-related gene activity [34]. Single-cell transcriptomic comparisons confirmed that astrocytes underwent greater genetic changes than any other brain cell type, including neurons [35, 36]. These changes are likely allowing astrocytes to contact and orchestrate the greater number of neurons in the expanded brain, as a single protoplasmic astrocyte in the human cerebral cortex contacts hundreds of thousands of synapses across multiple neuronal dendrites simultaneously [37]. Notably, engraftment of human astrocyte progenitor cells into mouse brains enhances animal learning, providing functional evidence that astrocyte properties can directly influence cognitive performance [38]. For decades, astrocytes have been perceived as a relatively homogeneous group of cells. However, it is now clear that astrocytes are phenotypically diverse, with their complexity increasing alongside the evolutionary expansion of brain size and neuronal network complexity. The “universal” fibrous and protoplasmic astrocytes are found in all mammals, residing in white and grey matter, respectively [39]. The primate brain contains a novel astrocyte subtype called interlaminar astrocytes. These cells reside in the first cortical layer and project long, up to 2 mm, protrusions throughout the cortical layers [40, 41]. Although their role is unclear, interlaminar astrocytes are thought to facilitate sufficient communication within the cortex [42]. It was shown that the processes of interlaminar astrocytes deteriorate in brain sections of Alzheimer’s disease patients, indicating their possible contribution to the efficient command of cognitive skills [43]. It is not entirely clear what molecular mechanisms drive the morphological specialization of astrocytes. It is being debated whether it is the same progenitor giving rise to distinct subtypes or the environmental cues are leading to obtain the specific morphology [44]. Likewise, the molecular basis of astrocytic evolutionary expansion is still under investigation.

Together, given the multifunctionality of astrocytes and their substantial advancements in primate brain, it is reasonable to ask whether these cellular changes can be associated with broader modes of neuronal network regulation. Established astrocytic functions, such as synaptic regulation, metabolic coupling, and calcium-mediated signaling, can influence neuronal activity beyond synaptic contacts. These mechanisms provide a plausible basis for astrocytes to integrate local signals and modulate neuronal populations across spatial and temporal scales. However, the extent to which such functions have changed during evolution, and whether additional molecular mechanisms contribute to this broader influence, remains open questions. In this context, ADEV-mediated signaling represents one candidate mechanism that could complement established astrocytic pathways.

## Extracellular Vesicles in the CNS

Extracellular vesicles (EVs) are lipid membrane-bound particles, released by virtually all cell types throughout the body, that serve as vehicles for intercellular communication. EVs can be classified by their origin, molecular composition and size. In general, they are divided into three major subgroups: exosomes, microvesicles, and apoptotic bodies. The smallest EVs, exosomes (~ 30–150 nm in diameter), are generated through the endosomal pathway, regulated by the endosomal sorting complexes required for transport (ESCRT) machinery and ESCRT-independent mechanisms. Microvesicles (100–1000 nm) are formed by direct outward budding and fission of the plasma membrane, a process dependent on cytoskeletal remodeling and calcium-dependent scramblase activity. Apoptotic bodies (500–2000 nm) are released during programmed cell death and are generally less relevant to physiological signaling in the healthy brain [45, 46].

In the CNS, where the cellular environment is exceptionally complex, EVs have emerged as a particularly important mechanism of local and long-range signaling. Constitutively produced by all major CNS cell subtypes, EVs drive intercellular communication in the brain and beyond [47–49].

### Molecular Cargo of Extracellular Vesicles in the CNS

The functional potential of EVs is determined by their molecular cargo, which reflects both the identity of producing cell and its physiological state at the time of vesicle release. CNS-derived EVs carry complex cargo consisting of lipids, proteins, and nucleic acids. Cargo composition is dynamically regulated by neuronal network activity, metabolic state, and pathological conditions [50, 51].

Lipids form the structural core of EVs. The EV membrane is enriched in cholesterol, sphingomyelin and phosphatidylserine, ensuring their mechanical endurance and efficient fusion with the recipient cells [52]. Beyond their structural roles, these vesicles transfer bioactive lipid species, for example ceramide, that can activate lipid-sensing receptors and respective pathways in the recipient cells [52].

At the protein level, CNS EV cargo constitutes transmembrane receptors, signaling molecules, metabolic enzymes and chaperones. Tetraspanins, especially CD9, CD63, and CD81, are enriched on EV membranes and serve both as biogenesis regulators and as canonical EV markers [46]. Cell-type-specific protein cargo signatures reflect the origin and state of the producing cell. Neuron-derived EVs are enriched in synaptic proteins, including GluA1, GluA2, synaptotagmin, and PSD-95 that, upon delivery, can impact dendritic spine formation and postsynaptic receptor organization [53, 54], while astrocytic EVs are enriched with neuroprotective agents such as ApoD [55] or heat-shock protein HSP70 [56].

Non-coding RNAs (ncRNAs) constitute the third major class of EV cargo, having important potential roles in gene regulation. Micro-RNAs (miRNAs) are the most abundant and the most thoroughly studied among ncRNA cargo species. miRNAs are small, ~22 nucleotide, non-coding RNAs that regulate gene expression in post-transcriptional manner by binding to complementary sequences in target mRNAs, inducing their degradation or translational repression [57]. EV packaging protects miRNAs from external ribonucleases [58], therefore they can exert a regulatory role in the recipient cells over timescales exceeding classical signaling, for instance by inducing epigenetic changes [59]. In addition to miRNAs, CNS EVs carry long non-coding RNAs, circular RNAs and even mRNAs, each of them with distinct regulatory potential [60–63].

### Functional Roles of the Extracellular Vesicles in the CNS

The molecular diversity of EV cargo translates into a broad range of functions. EVs not only participate in physiological intercellular communication, modulation of synaptic plasticity, and stress responses but are also involved in pathological processes. They act at distinct spatial scales, from local synaptic microenvironments to systemic distribution in the periphery, beyond the BBB.

EVs facilitate bidirectional communication between neurons and glial cells, including astrocytes, oligodendrocytes, and microglia. Astrocyte- and oligodendrocyte-derived EVs are taken up by neurons in an activity-dependent manner and provide them with metabolic support and neuroprotective cargo that contribute to maintenance of active circuits [47, 64]. Microglia-derived EVs modulate synaptic transmission, for instance by releasing endocannabinoids and regulating inhibitory signaling in rodent system [65]. Rat neuronal EVs carry activity-related synaptic cargo, signaling their current functional state to surrounding glia and contributing to receptor trafficking and organization of postsynaptic density [53, 66]. Apart from purely organizational functions, EVs can also serve as a means of cellular state communication. For instance, a recent work in a SHANK3 autism model found that EVs derived from mutant neurons could transfer altered excitability and maturation phenotypes to otherwise normal neurons, illustrating that EVs can carry biologically meaningful information about the state of their source neurons [67]. The continuous, multicellular EV-mediated dialogue supports the maintenance and adaptive remodeling of the local synaptic environment.

EVs’ cargo can also modulate synaptic plasticity, for instance through miRNA cargo. For example, in rodent primary hippocampal neuron cultures, neuron-derived EVs are enriched in miR-132-5p, miR-218-5p, and miR-690 that respectively, impact dendritic arborization and increases excitatory synapse clustering, synaptic vesicle organization and synchronous network activity of hippocampal neurons in vitro, demonstrating a direct EV-miRNA-driven mechanism for activity-dependent synaptic strengthening [68]. miRNAs can be also delivered from neurons to glia. Neuronal EV-derived miR-124a modulates GLT-1 expression in rodent astrocytes to improve glutamate clearance and stabilize synaptic transmission [69]. By delivering distinct miRNAs in a coordinated manner, EVs can extend the regulation of plasticity-related gene expression beyond the producing cell, contributing to wide-level modulation of network signaling.

EVs function as a stress response buffer. Upon conditions such as oxidative stress, hypoxia or excitotoxicity, both astrocytes and neurons upregulate EV production and enrich the cargos in antioxidative molecules, enzymes and heat-shock proteins, that are further distributed to vulnerable cells [56, 64, 70]. This EV-mediated protective mechanism extends and supports cell-autonomous protective responses and provides a means of limiting damage.

Moreover, EVs were shown to bidirectionally cross the BBB [71]. CNS-derived EVs carrying pathological cargo, for example amyloid-beta, tau, or alpha-synuclein to the peripheral circulation, enable diagnosis of Alzheimer’s and Parkinson’s diseases from patients’ plasma EV preparations [72, 73]. Conversely, EVs administered peripherally can deliver therapeutic cargo to the brain parenchyma, bypassing the impermeability of the BBB to large molecules. This possibility was shown in vivo by intranasal exosome delivery [74, 75]. Thus, the dual capacity of EVs as disease reporters and drug delivery vehicles makes them a target of translational neuroscience.

## Astrocyte-Derived Extracellular Vesicles (ADEVs): Molecular Cargo and Functional Roles

Astrocytes produce and release EVs constitutively, and ADEV content may differ in an activity- and stress-dependent manner [64]. Building on this general principle, ADEV cargo in particular is shaped by astrocyte reactivity state and regional identity [76]. As other EVs, ADEVs carry proteins, lipids, ncRNAs, and even whole organelles, together constituting a versatile signaling framework through which astrocytes respond to cues and modulate neurons and other CNS cells. Astrocytes are functionally diverse and so are EVs they produce. ADEVs exert neuroprotective, synapse-modulatory, neuroinflammatory, and under pathological conditions disease-propagating functions (Table 1). These functions are not fixed but rather dynamically adjusted in response to environmental cues, making ADEVs context-specific regulatory hubs.

**Table 1:** Summary of key studies on ADEV cargo and function discussed in this review, including information about EV source, cargo, reported effect and experimental model

Study	EV source	Molecular cargo	Reported biological effect	Experimental model
**Pascua-Maestro et al. **[55]	Astrocyte (ADEV)	ApoD	Prevents neuronal lysosomal damage under oxidative stress	Human neuron–astrocyte co-culture, in vitro; Rodent in vivo system
**Taylor et al.**[56]	Astrocyte (ADEV)	HSP70	Molecular chaperone protective role	Chick primary astrocytes, in vitro
**Hayakawa et al. **[78]	Astrocyte (ADEV)	Functional mitochondria	Restores respiratory capacity, promotes neuronal recovery	Rodent primary astrocytes and neurons, in vitro; Rodent model of ischemic stroke, in vivo
**Wang et al. **[83]	Astrocyte (ADEV)	Synapsin-1	Promotes neurite outgrowth	Rodent neuron culture, in vitro
**Gosselin et al. **[84]	Astrocyte (ADEV)	EAAT1/EAAT2 fragments	Suggested role in glutamate clearance beyond direct contacts	Rodent astrocyte-derived EV preparations, in vitro
**Jin et al. **[85]	Astrocyte (ADEV)	HEPACAM, ApoE ratio	Influences timing of neuronal morphological development/maturation	Rodent neuron culture, in vitro
**Li & Li **[80]	Astrocyte (ADEV)	miR-124-3p	Reduces neuronal apoptosis and oxidative stress	Rodent neonatal hypoxic–ischemic injury model, in vivo
**Pei et al. **[81]	Astrocyte (ADEV)	miR-190b	Suppresses autophagy-associated neuronal death under ischemic stress	Rodent ischemic stress model, in vitro
**Varcianna et al. **[82]	Astrocyte (ADEV)	miR-494-3p	Supports motor neuron survival by repressing SEMA3A pathway	Human, ALS model, in vitro
**Gamarra et al. **[87]	Astrocyte (ADEV)	Rps6 (ribosomal protein)	Modulates local translation in neurons, enhances synaptic integrity	Rodent, co-cultures in vitro
**Goetzl et al. **[72]**, Nogueras-Ortiz et al. **[88]	Astrocyte (ADEV), pathological state	Amyloid-beta, tau	Propagation of pathological proteins, neuronal damage	Human AD patient samples, in vitro
**Niu et al. **[73]	Astrocyte (ADEV), pathological state	Alpha-synuclein	Diagnostic potential	Human samples, in vitro

The protein cargo of ADEVs consists of structural and functional components. ADEV proteome is particularly enriched in metabolic enzymes, including members of the glycolytic pathway, glutamine synthetase, and fatty-acid binding proteins, which, when delivered, may provide recipient neurons with enzymatic machinery that supports local metabolism [64, 77]. Astrocytes upregulate EV production upon metabolic stress, glutamate excitotoxicity, hypoxia, and oxidative stress. In these conditions, ADEVs carry cargo proteins that are not synthesized in recipient cells and play a vital protective function [64]. For example, apolipoprotein-D (ApoD) is sent from astrocytes to neurons upon oxidative stress to prevent lysosomal membrane damage [55], while heat shock protein HSP70 delivery reduces neuronal apoptosis and improves mitochondrial function following oxidative challenge [56]. Upon damaging conditions, for example, in a mouse model of ischemic stroke, ADEVs have also been shown to transfer functional mitochondria to neurons in order to restore respiratory capacity and promote neuronal recovery [78]. Together, these mechanisms constitute a buffering system, activated in response of astrocytes to stressor cues across the EV diffusion territory.

Beyond their structural role, ADEV lipids also carry signaling functions. The ADEV membrane is enriched in sphingomyelin, cholesterol, and phosphatidylserine, ensuring mechanical stability and facilitating membrane fusion with target cells [52]. ADEV-transferred cholesterol is also required for synapse formation and the maintenance of dendritic membrane integrity to neurons, as shown in rat neuronal cultures in vitro [79].

Noncoding RNAs are among the most functionally consequential ADEV cargo components, as they exert post-transcriptional gene regulatory effects in recipient neurons that may persist over long timescales, far exceeding classical neurotransmitter signaling. EV packaging protects miRNAs from extracellular ribonuclease degradation, enabling functional delivery to target cells [58]. Several miRNA species have been specifically identified in ADEVs with validated neuronal targets. For example, in rodent injury models, ADEV-delivered miR-124-3p reduces neuronal apoptosis and oxidative stress after neonatal hypoxic–ischemic injury [80], miR-190b suppresses autophagy-associated neuronal death under ischemic stress [81], and miR-494-3p supports motor neuron survival by repressing SEMA3A-associated axonal destabilization pathways [82].

ADEVs can also modulate synaptic composition and transmission through distinct mechanisms. ADEVs mediate delivery of Synapsin-1 [83], thereby contributing to the synaptic organization. Further, fragments of glutamate transporters, EAAT1 and EAAT2, were found in the ADEVs preparations, suggesting the role of vesicles in regulation of glutamate clearance beyond direct astrocyte-neuron contacts [84]. Through the bidirectional regulation of the HEPACAM-to-ApoE ratio in ADEVs, these ADEVs influence the timing of neuronal morphological development and maturation [85]. Stimulation of human cultured neurons with ADEVs delivered from healthy astrocytes enhances neuronal morphology and firing rates, while treatment with ADEVs from reactive astrocytes exerts the opposite effects [86], demonstrating that the influence of ADEVs on neural circuits depends on the physiological state of the producing astrocyte. In a mouse model of Alzheimer’s disease, astrocyte-derived EVs carrying the ribosomal protein Rps6 were shown to modulate local translation in neurons and enhance synaptic integrity in vitro, providing direct mechanistic evidence that ADEV cargo can regulate protein synthesis in recipient neurons rather than only delivering pre-made proteins [87].

During pathological states such as Alzheimer’s disease, ADEVs have been implicated in propagating pathological proteins and stress signals leading to neuronal damage and network dysfunction [72, 88], highlighting the dual role of ADEVs as both protective and potentially harmful mediators within the CNS [77].

Together, ADEVs act as context-sensitive, multifunctional regulatory agents with homeostatic, synapse-modulating, and immunomodulatory roles. Their functional versatility, coupled with capacity to adapt release rates and cargo composition in response to physiological state, positions ADEVs as a dynamic communication mechanism extending astrocytic influence beyond direct cellular contact. Their capacity for diffuse, state-dependent molecular signaling beyond direct cell contacts may represent an evolutionarily significant feature of CNS organization that scales with the complexity of the astrocytes that generate it.

## Can ADEVs Modulate Neural Network Properties Beyond Synaptic Transmission?

Synaptic transmission is the primary, highly precise and locally restricted mode of neuronal communication [89], where neurotransmitter diffusion is tightly controlled by their uptake and degradation, and postsynaptic receptor fields are confined to nanometer-scale densities [90]. While this mode of signaling enables rapid and precise communication between individual neurons, it remains spatially limited to single synapses and cell-cell contacts. Further, this process is not tailored to deliver a shared, multi-molecule regulatory information to multiple neurons simultaneously. This raises the question of what mechanisms would enable precise coordination of larger neuronal populations. Several such mechanisms are already established, for example, long-range axonal circuits [91], neuromodulatory volume transmission [92], gap-junction-coupled astrocytic networks [30], and glymphatic fluid-mediated signaling [32]. However, these modes differ from EV signaling as they do not provide vesicular transport for delivering a defined, molecular cargo into the biosynthetic machinery of recipient cells. ADEVs provide a biophysically plausible candidate mechanism, as their lipid bilayer can protect multimolecule cargo from extracellular degradation during transit [58], and surface proteins can mediate targeted uptake by specific recipient cells [46]. ADEVs seem also a functionally feasible complementary system as their cargo enables the post-transcriptional modulation of the recipient cell, such as modulating  dendritic spine morphology and synapse formation in vitro, for instance via fibulin-2-mediated activation of TGF-β signaling in the rodent system [8].

We note that cellular and spatial specificity is currently the weakest-supported part of this model: candidate mechanisms such as surface-protein-mediated selective uptake by specific neuronal subtypes have been observed in vitro [76, 92] but have not been shown to produce spatially selective effects in intact tissue. Yet, this mode of action is distinguished from established non-synaptic and volume-transmission mechanisms by a specific, testable prediction: unlike the acute (seconds-to-minutes) physiological changes produced by diffusing neuromodulators or gap-junction-coupled ion-based signaling, ADEV-mediated modulation should result in transcriptional and translational changes in recipient neurons emerging over a timescale of hours to even days, consistent with the time required for gene expression and protein synthesis, as has been directly demonstrated in vitro for oligodendrocyte-EV-induced signaling changes in rat neurons [93].

If ADEV-mediated coordination does operate as proposed, it would convey potentially persistent regulatory effects across multiple recipient cells, theoretically extending astrocytic influence beyond the territory of a single astrocytic domain, though the actual spatial extent of this effect remains to be measured (see Table 2 “Evidence status summary” below).

**Table 2:** Evidence status summary

Component of proposed complementary model	Current evidence	Inference	Key alternative	Decisive experiment
**Astrocyte evolutionary diversification **[34, 37, 40]	Strong	—	—	Comparative transcriptomic and IHC/IF datasets
**Evolutionary changes in EV (quantitative changes) **[34]	Preliminary	May alter signaling capacity	Secondary consequence of cell state	Comparative EV release measurements
**Evolution changes ADEV cargo (qualitative changes)**	Unknown	Plausible	No functional change in cargo	Comparative ADEV proteome/RNA-seq
**EV spread over local cell–cell contact distances **[98, 99]	Limited for ADEVs	Possible	Uptake only within direct contacts	Endogenous lineage-specific EV tracing
**Selective neuronal uptake **[100]	Preliminary	Possible targeting	Non-specific uptake	Cell-type-resolved in vivo tracing
**Persistent neuronal effects **[8]	Evidence in vitro	Plausible	Transient effects	Longitudinal functional manipulation
**Network-level effect**	Unknown	Hypothesis	Conventional network mechanisms are sufficient	ADEV-specific perturbation + network recording
**Evolutionary contribution to cognition**	Unknown	Speculation	EV changes are incidental	Comparative functional-evolutionary analysis

### Evolutionary Perspectives: ADEVs and Neural Complexity

Brain evolution is marked by a progressive increase in the number and density of neuronal cells [1, 94]. This expansion creates a well-documented coordination challenge: axon diameter does not scale proportionally with the greater distance signals must travel in larger brains, so conduction delays increase with brain size [95], favoring spatially clustered, fast local circuits over uniformly fast long-range connectivity [96]. So far, the known solution to this problem, across species, is primarily myelination of high-priority fast pathways, not the addition of a slower signaling mechanism [97].

Whether the coordination challenge created by increasing brain size and evolutionary complexity was also met, in part, by an EV-mediated signaling is, at present, an open hypothesis rather than an established conclusion. We are not aware of direct evidence, in either direction, connecting brain-size scaling to reliance on EV-mediated communication specifically. We propose ADEV-mediated signaling as one candidate mechanism worth testing in this context, arising from the expanded astrocytic scaffold.

Before developing this model further, it is useful to state its evidence base explicitly. The evolutionary hypothesis rests on a nested set of propositions, each of which currently has a different evidentiary status (Table 2).

### A Proposed Framework for ADEV-Mediated Regulation of Neural Communication

**Fig. 1:**
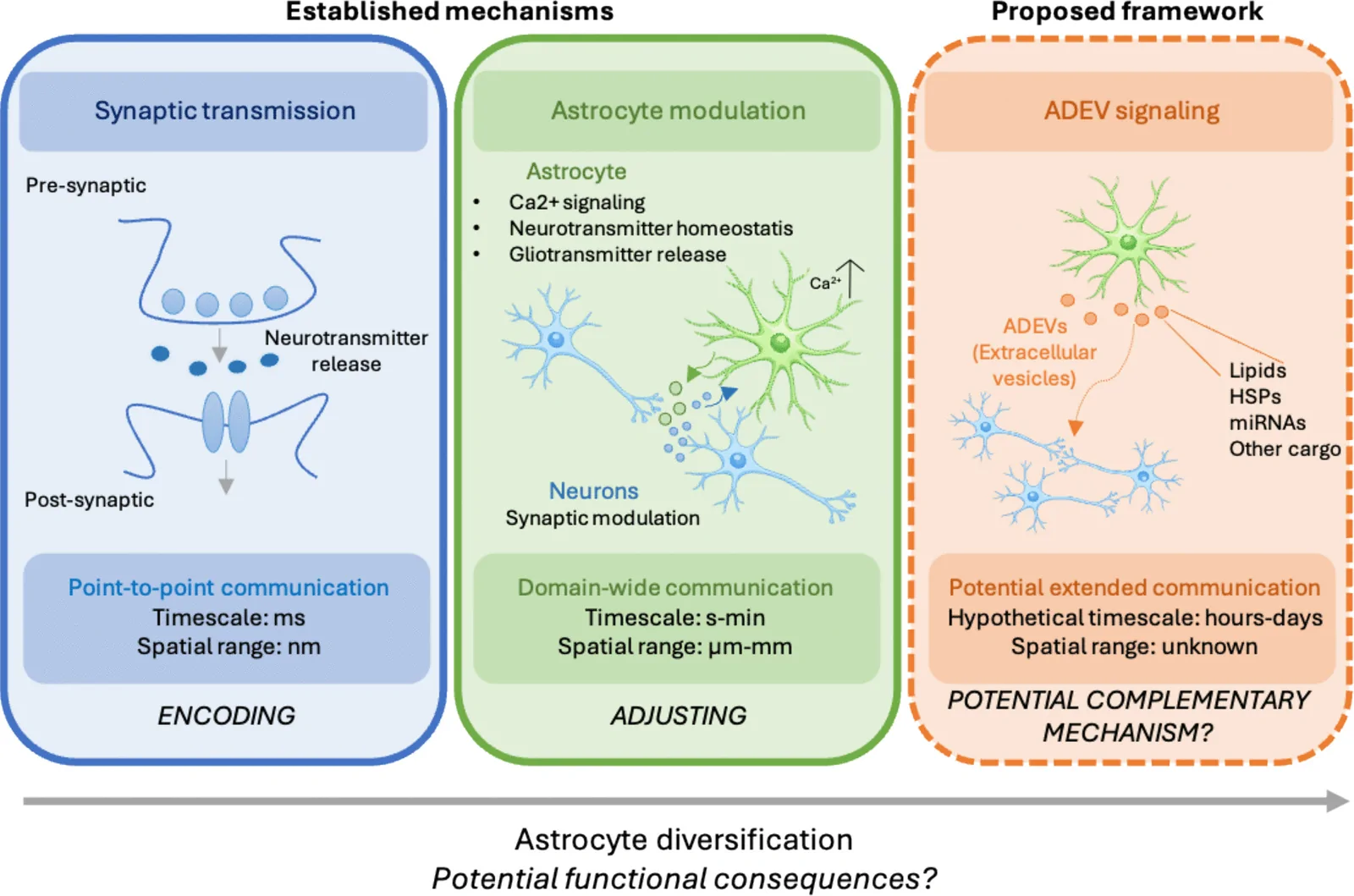
**Proposed framework for the potential contribution of astrocyte-derived extracellular vesicles (ADEVs) to neural communication**. Synaptic local communication is the primary mechanism of neuronal communication (blue). Astrocytes integrate and regulate neuronal function through established mechanisms including synaptic modulation, metabolic coupling, and calcium-dependent signaling (green). Astrocytes also produce extracellular vesicles containing proteins, lipids, and regulatory RNAs that can exert effects on recipient cells in experimental systems (orange). We propose that ADEV-mediated cargo transfer may represent a complementary mechanism through which astrocytes could influence neuronal cellular state beyond direct cellular contacts. The spatial range, cellular specificity, persistence, and network-level consequences of endogenous ADEV signaling in intact brain remain to be established. Dashed elements indicate proposed or unresolved components of the mechanism rather than experimentally established mechanisms

To place ADEVs in the broader context of brain communication, we propose a conceptual framework in which ADEV-mediated signaling represents a potential complement to established neuronal and astrocytic modes of communication (Fig. 1).

### ADEVs as a Proposed Complementary Mechanism of Neural Regulation

Direct in vivo evidence demonstrating astrocyte-to-neuron EV transfer at a spatial scale beyond individual astrocytic domains is currently lacking. The closest available experimental evidence demonstrates EV transfer in the reverse direction, from neurons to astrocytes, over distances on the order of hundreds of micrometers [101]. In addition, although astrocytes can alter EV production and cargo in response to cellular state [9, 102], it remains unknown whether such changes result in selective communication with defined neural populations in vivo.

If endogenous ADEV signaling does influence neurons beyond the immediate astrocytic territory, its functional distinction from other forms of non-synaptic signaling would likely arise from the nature and persistence of its molecular cargo rather than simply from its spatial range. ADEVs could, in principle, deliver combinations of molecules capable of modifying neuronal protein expression, metabolism, or synaptic properties over longer timescales than those associated with acute neurotransmitter or ion-mediated signaling. This possibility provides a testable hypothesis but does not establish that ADEV signaling constitutes a distinct network-level communication system.

The clearest evidence for astrocytes coordinating neuronal activity across larger spatial scales currently involves better established mechanisms, including gap-junction-coupled astrocytic networks and other forms of nonsynaptic signaling. Gap-junction-mediated coupling can extend across several astrocytes and over distances ranging from hundreds of micrometers to a few millimeters [30, 103, 104]. No comparable spatial scale has yet been demonstrated specifically for endogenous ADEV-mediated coordination. ADEV signaling should therefore be considered a candidate complement to these mechanisms rather than an established alternative to them.

Several alternative explanations exist for the hypothesis set out here that are not ruled out by current evidence and would complicate a straightforward reading of the proposed model. First, ADEV release may not be mechanistically independent of the faster astrocytic signaling. Because EV release from glial cells can itself be triggered by the same activity-dependent Ca^2+^ signaling that drives gliotransmission [64], it is possible that ADEV signaling is not an independent coordinating mechanism but rather a slow, downstream consequence of coordination that has already occurred via faster astrocytic signaling. Based on that, ADEV release could be perceived as a direct extension of astrocyte signaling activity rather than an isolated mechanism. Accordingly, the proposed framework does not require ADEV signaling to operate as an autonomous communication pathway and ADEVs may instead represent one downstream effector of established astrocytic sensing and signaling mechanisms. Distinguishing between these possibilities would require experimentally decoupling ADEV release from upstream Ca^2+^ signaling while preserving cargo delivery. Second, the observation that larger, more complex astrocytes produce more and/or differentially loaded EVs is also consistent with EV output being a passive consequence of increased membrane surface area and metabolic activity in a larger cell, rather than an independently functional or selected trait. This cannot be resolved based on the data available so far. Third, we note that established, independently sufficient contributors to primate cognitive expansion already exist, including increased neuron number and expanded morphology, synaptic density, and white matter connectivity. We do not claim that ADEV signaling is a key to explaining cognitive complexity, but rather propose it as an additional candidate mechanism whose explanatory contribution, if any, remains to be established relative to these better-characterized factors.

## Summary, Limitations, and Future Perspectives

The ADEV-mediated signaling mechanism we propose could, if confirmed, extend beyond local neuro-glia cross-talk. The proposed mechanism places ADEV-mediated signaling alongside established mechanisms of synaptic and astrocytic communication, while treating its physiological range, specificity, and contribution to network regulation as unresolved questions. Together it builds a conceptual framework in which ADEV signaling is a candidate contributor to complex brain communication, with regulatory scope hypothesized to scale alongside the evolutionary advancement of astrocytes and increasing brain size.

There are certain limitations for the functional framework proposed here. The comparative transcriptomics data that links astrocyte evolution with EVs requires further proteomic and functional analysis to support its mechanistic claims. The majority of data on ADEVs was obtained in a rodent system which is characterized by simplified astrocyte morphology and diversification; therefore, the direct extrapolation of this data should be cautious.

Direct experimental evidence for the evolutionary significance of ADEVs remains highly limited, partially because of specific technical challenges in the EV research field. These limitations include the lack of validated methods for tracking single vesicles and their cargo with defined cellular origin in intact brain tissue, the difficulty of assigning in vivo-released EVs to astrocytes versus other CNS cell types, and, in the scope of comparative studies, the restricted access to nonhuman primate (NHP) brain material. Nonetheless, several lines of indirect evidence are consistent with the framework we propose. Recent comparative transcriptomic data showed upregulation of EV-related genes in primate evolution [34] suggesting that the molecular machinery behind the production and release of EVs has been particularly activated in parallel with increasing astrocyte complexity. Moreover, the seminal engraftment study demonstrating that human glial progenitors enhance cognitive performance in mice [38] provides evidence that human-specific astrocyte properties, in general, can influence cognitive skills. Nevertheless, this evidence considers whole-cell transplantation rather than ADEVs specifically, and should not be read as direct support for ADEV-mediated circuit coordination. Direct loss-of-function evidence bearing on this model is still limited and remains context-dependent rather than astrocyte-specific. For instance, pharmacological blockade of EV release via nSMase2 inhibition has been shown to impair spatial and episodic memory under physiological conditions, consistent with a functional role for EV-mediated signaling in cognition [105]. However, the same manipulation improves cognitive outcomes in Alzheimer’s disease models, where it is thought to act by suppressing EV-mediated spread of amyloid-β pathology rather than removing a beneficial signal [106, 107]. These findings are not astrocyte-specific and do not isolate ADEV signaling from EV release by other CNS cell types, but they indicate that EV release causally affects cognitive function in a context-dependent manner.

Addressing these limitations requires major methodological advancements. One of the needs is the possibility of analysis of EV cargo in single vesicle resolution, ideally combined with its tracking in models as human/NHP cortical organoids or brain tissue slices. From today’s perspective, organoids provide the promising platform to test and track the impact of human ADEVs on neuronal maturation and network coordination and synchronization in species-specific context. Moreover, iPSC-derived models of human and NHP astrocytes can serve as a platform for comparative multiomics profiling of ADEV cargo complexity. It will be important to identify which molecular components of ADEV cargo are most prone to evolutionary changes and how they impact the neural network coordination. This would be especially interesting from the perspective of human-enriched neurological disorders, including autism spectrum disorder or schizophrenia, that are characterized by dysfunction of both astrocytes [108, 109] and EV composition and signaling [70, 110–112].

Taken together, existing data support a potential role for ADEVs as a candidate complementary mechanism capable of modulating neural cells, while their possible impact on neural networks remains to be experimentally established. Astrocytes have undergone substantial evolutionary diversification and produce molecularly diverse EVs. Whether these features are functionally coupled in ways that influence higher-level brain function remains unknown. Whether ADEVs themselves drive cognitive advancement, serve as a modulatory substrate for it, or play no significant role, remains an open question. We suggest astrocyte EV biology as a promising target for future investigation rather than an established driver of brain evolution. Testing the predictions outlined above will be necessary to determine how, and whether, ADEVs shape advanced brain function.

## Data Availability

No datasets were generated or analysed during the current study.
